# Complement Coercion: The Joint Effects of Type and Typicality

**DOI:** 10.3389/fpsyg.2017.01987

**Published:** 2017-11-24

**Authors:** Alessandra Zarcone, Ken McRae, Alessandro Lenci, Sebastian Padó

**Affiliations:** ^1^Department of Computational Linguistics, Universität des Saarlandes, Saarbrücken, Germany; ^2^Department of Psychology, Social Science Centre, University of Western Ontario, London, ON, Canada; ^3^Dipartimento di Filologia, Letteratura e Linguistica, Università di Pisa, Pisa, Italy; ^4^Institut für Maschinelle Sprachverarbeitung, Universität Stuttgart, Stuttgart, Germany

**Keywords:** sentence comprehension, complement coercion, thematic fit, semantic type, expectation generation

## Abstract

Complement coercion (*begin a book* →*reading*) involves a type clash between an event-selecting verb and an entity-denoting object, triggering a covert event (*reading*). Two main factors involved in complement coercion have been investigated: the semantic type of the object (event vs. entity), and the typicality of the covert event (*the author began a book* →*writing*). In previous research, reading times have been measured at the object. However, the influence of the typicality of the subject–object combination on processing an aspectual verb such as *begin* has not been studied. Using a self-paced reading study, we manipulated semantic type and subject–object typicality, exploiting German word order to measure reading times at the aspectual verb. These variables interacted at the target verb. We conclude that both type and typicality probabilistically guide expectations about upcoming input. These results are compatible with an expectation-based view of complement coercion and language comprehension more generally in which there is rapid interaction between what is typically viewed as linguistic knowledge, and what is typically viewed as domain general knowledge about how the world works.

## Introduction

In many theories of language processing, a comprehender’s expectations regarding upcoming input play a major role (Altmann and Mirkovic, 2009; Kuperberg and Jaeger, 2016). In these theories, expectations typically are triggered by multiple representational domains at different levels, and the integration of information across domains. These include information such as general semantic types (e.g., objects that denote entities vs. events) that are required by a predicate for its arguments (Jackendoff, 1997), and world knowledge about specific events and the participants involved in those events (Bicknell et al., 2010). The specific contributions of these types of information and their interplay remain open questions. In this article, we explore these issues from the vantage point of a particular and yet central phenomenon in sentence processing, complement coercion.

When reading a sentence like *The customer finished her burger*, people understand that a *customer* finished doing something with her *burger*, and that she probably finished *eating* it. Such constructions are examples of *complement coercion, logical metonymy*, or *enriched composition* (Pustejovsky, 1995; Jackendoff, 1997). Complement coercion arguably takes place whenever there is a type clash between a verb and its argument (Pustejovsky, 1995). In the example above, *finish* subcategorizes for an event-denoting object, but instead is combined with an entity-denoting object (*burger*). The meaning of the object is thus *coerced* into a predicate (*burger* is extended to mean *eating the burger*).

Complement coercions are an interesting test bed for theories of incremental language comprehension, sitting at the interface among syntax, semantics, and world knowledge (Hamm et al., 2006). The mismatch between their syntactic structure and their interpretation challenges compositionality by requiring the comprehender to generate a covert event by invoking lexical semantics and/or world knowledge. For example, interpreting *The customer finished the burger* involves knowing that customers *eat* burgers, whereas *The chef finished the burger* probably means *cooking* it.

A number of studies have identified processing costs for complement coercions when contrasted with sentences without type clash (*The journalist began / wrote the article*). These include McElree et al. (2001), Traxler et al. (2002), Pickering et al. (2004, 2005), Traxler et al. (2005), and Pylkkänen and McElree (2006, 2007), to name a few. Building on this rich body of studies, Traxler et al. (2005) and Frisson and McElree (2008) decomposed the sequence of operations involved in complement coercion into three steps. First, the verb–object type clash is detected as an obstacle to compositional interpretation. This causes a plausible covert event to be inferred. Following that, the inferred event sense is incorporated into the meaning of the phrase. Type clash was excluded as a source of cost, arguing that standard entity-to-entity metonymy, although involving a type clash, is less costly than entity-to-event coercion (but see Schumacher, 2011, 2013). Complement coercion typically yields reading time differences in later, post-target regions compared to non-coerced baselines, whereas semantic anomalies (*The journalist astonished the article*) increase reading times at the object (Pickering et al., 2004). It has been argued that recovering the covert event cannot be responsible for the coercion cost because it does not depend on the predictability of the covert event, and is still observed when the event is made explicit (Traxler et al., 2005; Frisson and McElree, 2008). These authors concluded that the cost arises from the additional construction of an event sense for the complement.

Complement coercions are a paradigmatic case to observe the joint role of semantic types and event knowledge in triggering expectations. In complement coercions, event-selecting verbs trigger expectations for objects of a specific type (*finish* →*cooking, eating*,…), sentential elements such as the subject and object contribute to expectations based on domain-general world knowledge (subject: *chef* →*roast, cook*; object: *burger* →*prepare, eat*), and the integration of these cues contributes to accessing a specific covert event relevant to the complement coercion (*The chef finished the burger* →*cooking*). In this article, we test whether expectations for an event-selecting verb following a subject and object in German verb-final constructions interact with semantic type in complement coercion, an aspect which has not been investigated before. We explore the time course of coercion by manipulating sentence content before the type clash is encountered and by investigating how the object’s type and subject–object real-world typicality interact.

### Expectation-Based Processing of Complement Coercion

Research has shown that language processing relies on rich knowledge about common events and their participants (see McRae and Matsuki, 2009, for a review). This generalized event knowledge is learned from first- and second-hand experience of everyday events (*washing a car, going to the doctor*,…) and is rapidly used during language comprehension. Usually, event-participant typicality is operationalized as *thematic fit* to the verb (event), which can be estimated either using human ratings, or from corpora by measuring the association between verbs and arguments (Lenci, 2011). Thematic fit can be influenced by context, such as other arguments (e.g., *check spelling* has a high thematic fit if the subject is a *journalist*, but low thematic fit if the subject is a *mechanic*, whereas the opposite is true for *check brakes*, Bicknell et al., 2010), and research has shown that speakers use and integrate syntactic, semantic, and world knowledge at each point during processing, and also to predict high-fit upcoming linguistic input (Altmann and Kamide, 1999; Hare et al., 2009; Milburn et al., 2016).

We hypothesize that sentence processing is guided not only by expectations about typical event participants, but also that expectations are influenced by the general semantic type of the arguments. Accounts of complement coercion have focused on the type clash between the selectional restrictions of the verb and the semantic type of the object, regardless of the object’s typicality. Importantly, we hypothesize that both expectations for a semantic type and expectations for a filler of an argument slot matching that type influence complement coercion processing, albeit at different levels of representation (the former more coarse-grained, the latter more fine-grained), and that they may rapidly interact as well. Verbs like *begin* trigger expectations for an event-denoting object, cueing high-typicality events depending on the context (*The surgeon started the operation*). If a low-typicality event, or an entity is encountered, expectations are violated and, in the case of a type mismatch with an entity-denoting object, a coercion operation is required to retrieve a (covert) event interpretation from generalized event knowledge.

Zarcone et al. (2014) showed that the subject and object in a complement coercion activate a covert event matching the cued scenario. In a self-paced reading experiment in German with an explicit event (*Der Konditor begann die Glasur aufzutragen/ The baker began the icing to spread*), event verb (*spread*) reading times were shorter after a high-typicality (*baker + icing*) versus a low-typicality (*child + icing*) subject–object combination.

In this study, we present a novel self-paced reading experiment exploring the interplay between type and thematic fit, testing whether the object’s semantic type and subject–object typicality interact to influence comprehension of event-selecting verbs.

## Experiment

Previous work has demonstrated the role of semantic type as a coercion trigger. Traxler et al. (2002) contrasted a coercion condition (*started the puzzle*) with three conditions without type clash. Thus, the same verb was combined with an event, matching its type restrictions (*fight*), and with an entity, clashing with its restrictions (*puzzle*). Neutral verbs that allow for entity and event objects (*saw*) also were used, as in (1).

(1) a. The boy **started/saw the puzzle** after school today.
b. The boy **started/saw the fight** after school today.

In self-paced reading, verb and object type interacted at the noun+1 position (*after*), with the longest reading times for the coercion condition. An eye-tracking study yielded an interaction between verb and object type in second-pass and total reading times at the noun. This was interpreted as evidence for the processing costs of complement coercions compared to constructions without type clash.

The present experiment also contrasts entity and event objects in combination with event-selecting verbs, but we introduce some key design modifications specifically aimed at disentangling the effects of type and thematic fit. The role of type has been investigated by manipulating the object’s type (entities vs. events), but the object’s typicality given the subject has never been manipulated before. Previous work has manipulated other factors such as the typicality of the subject–object–main verb combination (*The author was starting/writing/reading the book*, McElree et al., 2001), or how strongly constrained the covert event is (*The teenager began/read the novel* vs. *The waitress started/served coffee*, Frisson and McElree, 2008). In these studies, the object’s predictability has been held constant by using fairly plausible objects. Questions remain concerning how type and typicality of the object itself (operationalized as thematic fit to the subject–verb combination) interact during complement coercion, and if complement coercion costs accrue only for high-typicality cases (*book* is a highly typical object for *author*), or (within an expectation-based framework) if facilitation effects for expected type combinations (event-selecting verb + event-denoting object) can be observed only at the high-typicality range.

A second novel modification is that we conducted our experiment in German and exploited German word order. Example stimuli are in (2).

**Table:** 

(2)	a.	Das	Geburtstagskind	hat	mit	den	Geschenken	**angefangen**.
		The	birthday boy	PERF-AUX	with	the	presents	**begun**.
	b.	Das	Geburtstagskind	hat	mit	der	Feier	**angefangen**.
		The	birthday boy	PERF-AUX	with	the	party	**begun**.

English lacks an optimal way to compare reading times at the verb given different objects (unless with marked cleft sentences such as *It was the presents that the birthday boy opened*), whereas German sentences that feature perfect tense naturally place the main verb last. Our use of German syntactic structure enabled manipulating subject–object fit in a natural way prior to the verb. This allowed us to measure reading times for identical words across conditions at the critical aspectual verb, and to investigate the interaction between type and typicality directly at the verb. A type clash between an event-selecting verb and an entity object may happen as soon as the two are combined, regardless of which is presented first. Our experiment is the first to manipulate both type and thematic fit, using high- and low-fit entity- and event-denoting objects to test how type and typicality influences aspectual verb reading times. Typicality was dichotomized in our design (high vs. low typicality) as in previous studies on complement coercion (McElree et al., 2001; Frisson and McElree, 2008) and on generalized event knowledge (McRae et al., 1998; Rayner et al., 2004; Bicknell et al., 2010).

A further advantage of using German is that the deverbals are not formed by zero derivation and are capitalized (capitalization is exploited by German readers to facilitate processing, Hohenstein and Kliegl, 2013). Suffixation and capitalization help to disambiguate between German event nouns and the verbs from which they are derived, which is not the case for English zero-derivation deverbals such as *fight*, where only syntax (e.g., the presence of a determiner) disambiguates between the verbal and nominal form.

Finally, our experiment differed from previous studies in that only aspectual verbs were used. Aspectual verbs form a fairly well-defined class (e.g., *begin, finish*, Levin, 1993), and they clearly select for event-denoting objects. Previous studies have used a broader set of event-selecting verbs, including aspectuals and a mixture of non-aspectual, event-selecting verbs, such as psychological verbs (*enjoy, endure, savor*), plus others that do not clearly belong to any specific class (*attempt, expect, survive*). The psycholinguistic study in Katsika et al. (2012) and the computational study in Utt et al. (2013) suggest that non-aspectual event-selecting verbs differ from aspectual verbs both in their selectional preferences and in the processes, they trigger (inferential processes for non-aspectual event-selecting verbs, type clashes for aspectuals). We therefore restricted ourselves to aspectual verbs to avoid potential confounds arising from treating multiple verb classes as homogeneous. Piñango and Deo (2015) also argue that aspectual verbs allow for structured entity-denoting individuals as their objects, and that coercion effects are to be ascribed to the ambiguity of such structured individuals between an entity and an event reading rather than to a clash between the verb’s selectional preferences and the object’s type. Note however, that Piñango and Deo (2015) analyze the full range of the simple transitive uses of aspectual verbs, both in their eventive sense (e.g., *The customer finished her sandwich*) and in their non-eventive sense (e.g., *A little porcelain pot finished the row)*, whereas the scope of our work is limited to the eventive sense of aspectual verbs.

A type-based account predicts only an effect of semantic type on the verb reading times. The availability of a compatible covert event is excluded as a source of cost (Traxler et al., 2005; Frisson and McElree, 2008), and thus the cost of coercion should be observed regardless of the fit between subject–object or among the subject–object–verb triplet. Our study differs from previous studies of complement coercion primarily because we used German subject–object–verb sentences rather than the English subject–verb–object sentences used in the majority previous studies on this topic. Thus, in our study, the critical region of interest is the aspectual verb, whereas in previous studies, it was the post-verbal object. In other words, in subject–object–verb studies, the primary concern is how the object is expected and integrated given that a (event-selecting vs. non-event-selecting) verb has already been read. In our study, the primary concern is how the aspectual verb is expected and integrated given that a comprehender has already encountered a subject and a (high-fit vs. low-fit event or entity) object. The sentence structure also entails a slightly different way of thinking about (thematic) fit. Thematic fit typically is discussed and measured as the fit of an agent, patient, instrument, or location with a type of event (verb). In the present research, fit is considered primarily with respect to a subject and an object, which, in our case, corresponds to how an agent and patient fit together semantically even though the main verb has not been encountered.

It is important to lay out clearly how expectations relate to the sentences used in our experiment. Sentences unfold dynamically over time, and therefore expectations evolve dynamically over time as a sentence unfolds. The pattern of expectations thus differs by sentence structure, that is, by the input that a comprehender has experienced at a specific point in time. In a typical coercion study in English, a sentence such as “The author began the book” might be presented. When a participant reads “author” (in this case, out of context, as in many psycholinguistic experiments), she might implicitly expect an upcoming type of action that authors typically participate in, such as writing. Another way to state this is that some set of potential continuations related to authors might be pre-activated to different degrees (Altmann and Mirkovic, 2009; Kuperberg and Jaeger, 2016). When “began” is read, due to type constraints, readers might expect some type of event to be upcoming in the linguistic stream, such as “writing” or “reading”. A comprehender might, to a lesser extent, expect an entity such as book to occur somewhere in the sentence because authors do tend to begin writing and reading books and similar entities such as articles.

The present study differed in important ways because the German sentences that participants read unfolded in a critically different order. We used sets of sentences (presented using English glosses here) that follow this format:

(3) a. *[High Fit]*. The birthday boy has with the presents/party at once started...
b.*[Low Fit]*. The birthday boy has with the soup/shift at once started...

All of our sentences begin with an animate subject. Given the animate subject, some kind of verb must directly follow in German main clauses (”V2 position”). Our sentences provide an auxiliary verb (“has”) following the subject.

Next, a number of potential syntactically and semantically reasonable continuations exist following “The subject has (with) the”. After the verb, in the so-called “Mittelfeld” of the topological model of German syntax, word order is largely free, in particular in the absence of discourse context (Wöllstein, 2010). However, given “The (animate) subject has (with) the”, and with a case marking on the determiner that is consistent with the accusative case of direct objects, there is arguably a very strong expectation of an upcoming object. Thus, readers most likely expect objects that have a high fit with the subject, such as “presents” or “party” given “birthday boy”, even without a verb being specified at this point. If subject–object fit influences expectations and comprehension generally, then reading times would be predicted to be shorter for objects that tend to appear in the same scenarios as the subject. Note that there is no strong reason to think that readers would be biased toward entities versus events. No main verb has been encountered at this point, so type is not particularly relevant to comprehending the sentence, and the strongest constraint should be how well the subject and object go together. Finally, note that because event nominals tend to be longer than entity nominals in German, longer reading times for event objects might be observed (see below).

The adverbial (“at once”) follows the object. While the adverbial between the object and the aspectual verb is being read, there may potentially be spillover effects due to differences in subject–object fit. The critical issue concerns what happens when people read the aspectual verb in this sentential context. Because the position of the adverbial in the Mittelfeld is very flexible (Wöllstein, 2010), the sentence could continue in a number of ways. However, having read a subject, an object, and an adverbial, there is bound to be a strong expectation for an upcoming transitive verb. If semantic type is the only factor that drives expectations for a verb, then one would predict shorter aspectual verb reading times for events than for entities regardless of subject–object fit (i.e., a main effect of type, with no main effect of fit nor an interaction). That is, one might predict that as long as any aspectual verb follows an event versus an entity object, then reading times should be faster following event objects because an aspectual verb is more highly expected than a content verb.

On the other hand, it might be the case that expectations at the point at which the verb occurs depend on both type and subject–object fit. The high fit sentences provide a suitable environment for expectation-driven processing because the sentence elements cohere to this point in that strong relations between the subject and object promote expectation generation. Therefore, given a subject like “birthday boy” and event object like “party”, an aspectual verb such as “started” would be expected, although other continuations certainly are possible. On the other hand, the combination of “birthday boy” and “presents” should lead to expectations for content verbs such as “opened” or “loved”. In this way, the high-fit combination of the subject and object would lead to a strong effect of type when the aspectual verb is read.

The case of low subject–object fit differs. There is no established general relation in memory between “birthday boy” and either “soup” or “shift”. The lack of such relations between the subject and object produces an environment that promotes weaker, broader generation of expectations (see Kuperberg and Jaeger, 2016, for an extended discussion). This might lead to two possible results. The first is that because no clear schema or scenario is produced by low-fit subject–object combinations, the influence of type might be absent. This could result from weak expectation generation perhaps in combination with the difficulty of integrating the low-fit subject–object–verb triplet. The second possibility is that an effect of type might be found, but it might be muted relative to the high-fit sentences because high-fit as compared to low-fit subject–object combinations lead to expectations for verbs that are clearer or stronger.

### Method

#### Participants

Forty-eight Universität Stuttgart students (20 females; age: *M* = 23 years; *range* = 18–32) were paid to participate. All participants were native German speakers and had normal or corrected-to-normal visual acuity.

#### Materials

The materials for the self-paced reading experiment were based on two norming studies and an expert annotation study (see Appendix A for the stimuli used in the self-paced reading experiment). In **Norming Study 1**, participants produced objects for 25 sentence templates (*Der Student hat mit dem/der _____ angefangen*/ *The student has with the _____ begun.*) on Amazon Mechanical Turk (AMT). Six native German speakers provided plausible objects. All sentence templates contained a verb referring to the temporal structure of the event (*anfangen [start], aufhören [finish], beginnen [begin], vertagen [postpone], weitermachen [continue]*). Space was provided for five responses and no time limit was imposed. We chose 4 objects for 21 sentences, from the objects provided most frequently, weighted by production rank. We selected 21 out of 25 sentences where at least two entity and two event nouns were among the most frequently produced responses for them (*The student has with the essay_entity_/book_entity_/study_event_/exam_event_ begun*).

In an **Expert Annotation Study**, three linguists (native German speakers) annotated the 84 (4 × 21) object nouns as event-denoting, entity-denoting, or ambiguous. We selected the 40 objects with the highest agreement scores (2 objects × 20 sentence templates), discarding ambiguous ones. Weighted α (Krippendorff, 2013) for the selected nouns (weighting entity-event disagreements more highly than entity-ambiguous and event-ambiguous disagreements) was 0.71 (good agreement).

Forty high-fit sentences were constructed. An adverb was inserted between the object and sentence-final verb (past participle) to diminish possible spillover effects into the verb region, and the sentence continued after the aspectual verb, as in (4). The adverbs were not included in the completion study, but we chose quite generic adverbs that were unlikely to influence the event interpretation in the test sentences in any systematic fashion.

**Table:** 

(4)	a.	*[entity]*	Das	**Geburtstagskind**	hat	**mit**	**den**	**Geschenken**	sofort	**angefangen**,	obwohl	seine	Mutter	nicht da war.
			The	**birthday boy**	PERF-AUX	**with**	**the**	**presents**	at once	**started**,	although	his	mother	wasn’t there.
	b.	*[event]*	Das	**Geburtstagskind**	hat	**mit**	**der**	**Feier**	sofort	**angefangen**,	obwohl	seine	Mutter	nicht da war.
			The	**birthday boy**	PERF-AUX	**with**	**the**	**party**	at once	**started**,	although	his	mother	wasn’t there.

Forty low-fit sentences were constructed by assigning the high-fit objects to another sentence template that used the same verb. This template in turn was paired with the high-fit objects from the first template as its low-fit objects.

**Table:** 

(5)	a.	*[entity]*	Das	**Geburtstagskind**	hat	**mit**	**der**	**Suppe**	sofort	**angefangen**,	obwohl	seine	Mutter	nicht da war.
			The	**birthday boy**	PERF-AUX	**with**	**the**	**soup**	at once	**started**,	although	his	mother	wasn’t there.
	b.	*[event]*	Das	**Geburtstagskind**	hat	**mit**	**der**	**Schicht**	sofort	**angefangen**,	obwohl	seine	Mutter	nicht da war.
			The	**birthday boy**	PERF-AUX	**with**	**the**	**shift**	at once	**started**,	although	his	mother	wasn’t there.

When crossing the items to construct the low-fit sentences, we ensured that the low-fit objects were never elicited for those subject–verb combinations (*Suppe* [*soup*] and *Schicht* [*shift*] were never elicited for *Das Geburtstagskind + angefangen*). Entities and events did not differ in their mean log frequency in the CELEX German word frequency list (Baayen et al., 1993: 1.49 for entities and 1.38 for events; Wilcoxon rank sum test: *W* = 881, *p* = 0.438). They differed in number of letters (entities: *M* = 13 letters; events: *M* = 15 letters; *W* = 489, *p* = 0.003). This is unavoidable because event nominals in German often are formed by suffixation.

To check that the low-fit sentences were plausible, we conducted a plausibility rating study on AMT. In **Norming Study 2**, 16 native German speakers rated the 80 sentences on a 1 to 5 scale, along with 64 nonsensical fillers, reaching high agreement (α = 0.72). We provided examples and definitions for points 1, 3, and 5 of the scale (5 = very plausible, e.g., *A hunter shot a deer*; 3 = possible in principle but not very plausible, e.g., *The child held a lecture*; 1 = impossible, e.g., *A deer shot a hunter*). Mean rating was 4.41 (*SD* = 0.58) for high-fit sentences, 2.92 (*SD* = 0.56) for low-fit sentences, and 1.58 (*SD* = 0.56) for non-sensical fillers. The plausibility ratings for the low-fit sentences were significantly higher than for non-sensical fillers (*W* = 1140, *p* < 0.001) and significantly lower than for the high-fit sentences (*W* = 1522, *p* < 0.001). Thus, the low-fit sentences were less typical but made sense (someone can work on their birthday). Furthermore, plausibility ratings for sentences with event objects (*M* = 3.84, *SD* = 0.89) were non-significantly higher than for those with entity objects (*M* = 3.48, *SD* = 0.96, *W* = 618, *p* = 0.08).

#### Procedure

Four lists of 92 sentences (5 high-fit/entity, 5 high-fit/event, 5 low-fit/entity, 5 low-fit/event, 72 fillers) were created. Participants saw each subject–object combination at most once. Sentences sharing a subject were assigned to different lists. We used a one-word-at-a-time moving-window self-paced reading task. Each sentence was followed by a yes/no comprehension question. Participants took a break after each third of the items.

### Results

All participants scored better than 85% correct on the comprehension questions (*M* = 95%, *SD* = 0.05). Items for which participants incorrectly answered the comprehension question and reading time outliers (>2.5 *SD*s from the mean per region) were excluded from analyses (5% datapoints at the object, 5% at the object+1 (adverb) region, 5% at the verb and 7% at the verb+1 region).

We examined type and typicality effects in each region using a generalized mixed-effect regression model (dependent variable: log-transformed reading time; random effects: item, participant; fixed effects: type, typicality). We started with a maximal model including the random intercepts and slopes for all fixed effects (Barr et al., 2013), dropping the random intercept/slope correlation whenever the maximal model would not converge. We then included word length, log-transformed word frequency, reading time at the previous region, and presentation order as covariates, using forward step-wise model comparisons, as recommended by Baayen and Milin (2010, p.19), who state that, “Across many experiments, we have found that including variables such as TRIAL and PRECEEDING RT in the model not only avoids violating the assumptions of linear modeling, but also helps improving the fit and clarifying the role of the predictors of interest.” Word length significantly improved model fit at all four analyzed regions. Word frequency significantly improved model fit at the object and the verb regions. Reading time at the previous region significantly improved model fit at the object, object+1, and verb region, and the *p*-value was 0.054 at the verb+1 region. Presentation order significantly improved model fit at all four regions. (The statistics for the variables of interest when including all four covariates are presented in the text and **Table 1**. Statistics when excluding the four covariates appear in Appendix B). Finally, we obtained *p*-value statistics using the lmertest package, where *p*-values and degrees of freedom are calculated for the *t*-test based on Satterthwaite approximation for denominator degrees of freedom.

**Table 1:** Reading times (ms) and mixed-effect regression statistics.

		Region			
		Object	Object+1	Verb	Verb+1
		mit der Feier	sofort	angefangen	obwohl
		with the party	at once	started	although
**Reading Times (ms)**			
High-fit event		655	644	736	473
High-fit entity		642	656	819	508
Low-fit event		710	682	806	505
Low-fit entity		667	693	802	520
**Mixed-effects Regressions**			
Type	*t*	-1.86	-1.26	0.39	-3.34
	*p*	0.070	0.206	0.694	0.001
Fit	*t*	-2.06	-2.20	0.41	-2.21
	*p*	0.047	0.028	0.685	0.035
Interaction	*t*	-0.67	0.46	-1.94	-0.59
	*p*	0.504	0.640	0.054	0.558

Mean reading times and the associated mixed-effects regressions are presented in **Table 1**. Not surprisingly, no significant effects were found before the object region because across conditions, the sentences were identical up to that point.

#### Object Region (*mit der Feier*)

Reading times for high-fit objects were shorter than for low-fit objects (*t* = -2.06; *p* = 0.047). Reading times for entities were marginally shorter than for events (*t* = -1.86; *p* = 0.070). This marginal difference in reading times was most likely due to the event objects being longer (in terms of number of letters) than the entities. As stated above, event nominals in German often are formed by suffixation, and thus tend to be longer. There was no interaction (*t* = -0.67; *p* = 0.504).

#### Object+1 Region (*sofort*)

The effect of subject–object fit continued into this region (*t* = -2.20; *p* = 0.028). The effect of type (*t* = -1.26; *p* = 0.206) and the interaction (*t* = 0.46; *p* = 0.640) were non-significant.

#### Verb Region (*angefangen*)

At the critical verb region, where a type clash was expected, reading times after high-fit events were shorter than for each of the other three conditions. This produced an interaction between object type and thematic fit (*t* = -1.94; *p* = 0.054). There are two ways to think about the interaction (the influence of fit for each level of type, and the influence of type for each level of fit), and we present a pair of comparisons for each. Verb reading times were shorter after high-fit compared to low-fit events (*t* = -1.98; *p* = 0.053), but the effect of thematic fit was non-significant for entities (*t* = 0.43; *p* = 0.668). Verb reading times were shorter after high-fit events compared to high-fit entities (*t* = -2.19; *p* = 0.036), but the effect of type was non-significant for low-fit objects (*t* = 0.32; *p* = 0.749). This pattern of simple effects shows that the shortest verb reading times were found after high-fit events, the condition in which both type and subject–object fit preferences of the verb are met.

#### Verb+1 Region (*obwohl*)

There was a main effect of type (*t* = -3.34; *p* = 0.001) and thematic fit (*t* = -2.21; *p* = 0.035), without an interaction (*t* = -0.59; *p* = 0.558).

### Completion Study

Our general hypothesis is that reading times are related to probabilistic implicit expectations generated during on-line comprehension. Many studies have used sentence completions as a window into readers’ expectations. Therefore, to further investigate people’s expectations, we collected sentence completions from a separate group of participants (22 native German speakers) for verbs given our target sentences up to the word preceding the verb. The completion study was conducted on the Prolific Academic web platform, where participants were asked to complete each sentence with a verb.

It should be noted that although off-line completions often mirror on-line reading times, the task demands differ in important ways. First, participants are not under time pressure in a completion task so that they have time to read each sentence fragment and decide on a suitable completion. In contrast, natural reading is fast, providing little time for generating expectations. Second, participants must produce explicit completions in a sentence completion study, whereas expectation generation is implicit in normal language comprehension. Finally, specific to our study, we measured production of (and reading times for) verbs like *begin* and *end*, rather than verbs with greater and more specified semantic content. Therefore, we were interested to investigate how the sentence completions would relate to reading times at the aspectual verb.

We first calculated the proportion of trials on which participants produced the precise aspectual verb that was used in the reading time experiment (e.g., 0.23 if the specific aspectual verb was provided by 5 out of 22 participants). The completion statistics (see **Table 2**) are consistent with three of the four comparisons conducted on the verb reading times (comparisons between proportions were conducted using chi-square tests). The completions match the shorter verb reading times following high-fit events versus high-fit entities (11% vs. 5%, *z* = 3.31, *p* = 0.0009). They also are consistent with non-significantly different verb reading times following high-fit versus low-fit entities (5% vs. 7%, *z* = 1.50, *p* = 0.134). The off-line completions also mirror the lack of a difference between low-fit events and low-fit entities (9% vs. 7%, *z* = 0.96, *p* = 0.339). The influence of subject–object fit was found in the reading times for high-fit versus low-fit events, but this was not found in the completions (11% vs. 9%, *z* = -1.01, *p* = 0.315). Finally, note that in all cases, the precise aspectual verb was produced at most only 11% of the time.

**Table 2:** Proportions from the completion study.

Condition	Target verb	Aspectual verb
High-fit event	0.11	0.79
High-fit entity	0.05	0.33
Low-fit event	0.09	0.79
Low-fit entity	0.07	0.34

These results are interesting because they diverge from the reading time results for one specific comparison. In reading times, the fit or coherence of the subject and object resulted in shorter reading times at the aspectual verb when it was preceded by high-fit as compared to low-fit events. In contrast, in the completions, the influence of subject–object fit was diminished. We hypothesized that high-fit sentences might result in shorter reading times at the verb because it is easier to combine the subject and object semantically (in terms of, e.g., constructing a coherent situation model), thus promoting expectation generation. However, this did not appear to influence the results substantially when participants were given time to read the sentences, and were required to produce a verb as a continuation.

This lack of an influence of subject–object fit on the completions also is evident in the overall aspectual verb completions. We calculated the proportion of completions in which participants produced any aspectual verb (rather than specifically the verb that was used in the self-paced reading study). Completion proportions were 0.79 for both high- and lows-fit events. These were much greater than for high-fit (0.33) and low-fit entities (0.34), which were virtually identical.

Overall, when people have time to read a sentence fragment and think about it, and are asked to explicitly produce a verb, the probability with which they produce an aspectual verb is driven primarily by object type. In contrast, during speeded reading, the ease with which people read an aspectual verb following a subject–object combination is influenced by both object type and the ease with which the subject and object can be combined.

## Discussion

Our experiment is the first to enable demonstrating that thematic fit and type interact rapidly during complement coercion processing. The effect of subject–object fit began at the object, and continued through the verb+1 region. The effect of type began at the verb region, where the type clash is assumed to occur, in the form of an interaction with subject–object fit. At the verb+1 region, there were main effects of type and thematic fit.

Note that we obtained a number of expected significant effects in this experiment even though our reading time study used 20 items (five items per condition for each participant) and 48 participants in a two by two design, and that the key interaction corresponded to a *p*-value of 0.054. We used fewer items than in a number of similar experiments because one issue with conducting studies on complement coercion is that there exist a relatively small number of truly aspectual verbs. Particularly in a case like the present experiment in which the critical reading time measures occur at the aspectual verb, one does not wish to use the same verbs a large number of times. Doing so might create a less sensitive design due to repeating verbs. Therefore, we believed that 20 items would strike a balance given these constraints. It is likely, however, that including a greater number of items would have strengthened our inferential statistics.

The effect of type is in principle compatible with a type-based approach to complement coercion (Pustejovsky, 1995), and with previous studies showing coercion costs at the post-target region, interpreted as evidence for type shift accommodation (Traxler et al., 2002, 2005). However, our design enabled investigating what happens before the type clash. The facilitation effect of both type and fit that we observed at the aspectual verb supports context-driven expectations that are due to the joint influences of type and subject–object fit. The interaction of type and typicality was not investigated in previous research (e.g., Traxler et al., 2002), which used relatively uniformly plausible objects given the context.

Previous accounts such as Traxler et al. (2005) and Frisson and McElree (2008) excluded the availability of a compatible covert event as a source of cost. A cost for coercion should thus be observed regardless of the fit between subject and object. Our study yielded a cost for the coercion combinations but, crucially, only at the high-typicality range, whereas the low-typicality range was not taken into consideration in previous studies. Our account suggests that the processing of coercion might be influenced not only by the object type, but also by thematic fit, and predicts facilitation when expectations are met, thus predicting a difference between entities and events at the high-typicality range compared to low-typicality combinations. Whether a covert event is more or less available for a given subject and object combination, we investigated expectations for an aspectual verb, which is more expected in combination with an event-denoting object.

Verbs’ biases for syntactic structure have been shown to interact with rich world knowledge cued by context, resulting in incremental expectations throughout sentence processing (Trueswell et al., 1994; Hare et al., 2003; Elman, 2011). Expectations can involve both general semantic type (event vs. entity) and more fine-grained knowledge about specific participants. Our experiment demonstrates that both kinds of probabilistic expectations are active in complement coercions, a fact that cannot be explained by a strictly type-based approach. In contrast, an expectation-based view of complement coercion predicts that event-selecting verbs are read more quickly after event objects *if* the object coheres with the event knowledge cued by the subject.

When a subject is combined with an object denoting a typical event (*birthday boy + party*), then an event-selecting verb such as *started* is more highly expected; after a high-fit entity, an event-selecting verb is an unexpected continuation, whereas a content-loaded action verb presumably is more strongly expected (*birthday boy + presents* →*open*). When the subject is combined with a less typical object (event or entity), no clear expectation for a verb results, leading to relatively long reading times in the self-paced reading study for an event-selecting verb. Event-based knowledge also provides an ideal candidate for the context-sensitive retrieval of the covert event as a high-typicality event involving the subject and the object of the complement coercion (Zarcone et al., 2014).

Arguing for an interaction of type and typicality in sentence processing does not entail assuming that these are qualitatively distinct kinds of information. Instead, we regard them as two aspects of the same generalized knowledge about events, with semantic types representing a higher level of abstraction over specific event arguments. For instance, Zarcone et al. (2015) showed that corpus-based co-occurrence statistics regarding typical event participants can model both type and typicality effects in coercion sentences.

Previous work by Zarcone et al. (2013) employed a computational model of thematic fit (without an explicit representation of type) to reproduce patterns of behavioral results on complement coercion previously explained in terms of semantic type. This raises the question of whether thematic fit only can account for our experimental data. However, semantic type, as a higher-level kind of semantic constraint, may still be necessary to account for the difference between high-fit entities and high-fit events. In a similar fashion, research on standard metonymy (e.g., *The espresso wanted to pay*, Schumacher, 2011, 2013) has shown that standard metonymy does have an inherent type-driven processing cost that can not be canceled by providing supporting context (making the aspectual noun high-fit with previous context).

An expectation-based view of complement coercion, integrating type, typicality, and their interactions as natural components of incremental language interpretation, treats complement coercion not as a special case, but highlights that it shares central properties with “normal” cases of online language processing, notably being incremental, efficient, and fast.

## Conclusion

We presented a novel experiment demonstrating the interaction of type and typicality during comprehension of complement coercions as predicted by an expectation-based approach. We exploited German word order to compare the same target region across conditions. As in Traxler et al. (2002), we also obtained an effect of object type at the verb + 1 condition. Unlike previous studies, however, we included typicality as a factor to investigate the interaction between type and typicality. This interaction appeared directly at the verb region, demonstrating combined influences of generalized real-world event knowledge and verb-driven type preferences during language comprehension.

In many theories of language processing, the specific manner in which object type is associated with a verb is a purely linguistic matter, presumably being stored in the lexicon. On the other hand, real-world knowledge of the relations between the subjects in our sentences, which referred to kinds of people, and the objects, which referred to kinds of non-living things or real-world events, is definitely outside of the lexicon and is part of people’s broader knowledge about the world. Under this type of view, therefore, as Myachykov and Scheepers state in the description of this research topic, “resource-efficient and successful cognitive processing requires integration of information across multiple sources, modules, or even cognitive domains.”

## Ethics Statement

As per German law, psycholinguistic studies do not require consent by an ethics commission. All subjects were adults. All subjects were informed in writing about their rights following the recommendations of ZENDAS (Zentrale Datenschutzstelle der baden-württembergischen Universitäten). All subjects gave written consent in accordance with the Declaration of Helsinki. No subjects took advantage of their right to have their measurements deleted.

## Author Contributions

AZ drove the project in terms of designing the experiment, conducting the experiment, analyzing it, and writing the manuscript. KM, AL, and SP participated in designing the experiment, conceptual development, interpreting the data, and writing the manuscript.

## Conflict of Interest Statement

The authors declare that the research was conducted in the absence of any commercial or financial relationships that could be construed as a potential conflict of interest.
