# Supplementary material for: Complement Coercion: The Joint Effects of Type and Typicality
Source: Front Psychol. 2017 Nov 24;8:1987. doi: 10.3389/fpsyg.2017.01987 (PMC5705615; doi:10.3389/fpsyg.2017.01987)
Supplement: Supplementary file 1 [file Data_Sheet_1.pdf]

Appendix A

The translations of the experimental materials aim to remain as faithful to the German sentences as possible while rendering comprehensible English sentences. Personal pronouns mirror German grammatical gender. The primary difference with German is the word order (verb second in the main clause / verb final in the subordinate clause). Note that the materials are in colloquial/spoken German, where Perfekt is the usual past tense (*hat angefangen*). Thus the sentences are in the past (*started.... wasn't*), although the tense sounds similar to the English present perfect.

Each sentence subject was matched with two high-fit objects (one entity-denoting, one event-denoting object) and two low-fit objects. The high-fit objects for each odd-numbered item were used as low-fit objects for the following even-numbered item, and the high-fit objects for each even-numbered item were used as low-fit objects for the preceding even-numbered item.

1. [high] Das Kind hat mit dem Spielzeug / mit der Schlägerei ohne zu klagen aufgehört, weil es sehr müde war.

   The child has with the toy / scuffle without complaining finished, because it was very tired.

   [low] Das Kind hat mit dem Medikament / mit der Therapie ohne zu klagen aufgehört, weil es sehr müde war.

   The child has with the medicine / therapy without complaining finished, because it was very tired.

2. [high] Der Patient hat mit dem Medikament / mit der Therapie ohne zu klagen aufgehört, weil er Kopfschmerzen hatte.

   The patient has with the medicine / therapy without complaining finished, because he had a headache.

   [low] Der Patient hat mit dem Spielzeug / mit der Schlägerei ohne zu klagen aufgehört, weil er Kopfschmerzen hatte.

   The patient has with the toy / scuffle without complaining finished, because he had a headache.

3. [high] Der Autor hat das Buch / die Buchvorstellung endlich begonnen, um nicht in Verzug zu geraten.

   The author has the book / the book presentation finally begun, to stay on schedule.

   [low] Der Autor hat das Bier / die Säuberung endlich begonnen, um nicht in Verzug zu geraten.

The author has the beer / the cleaning finally begun, to stay on schedule.

4. [high] Der Braumeister hat mit dem Bier / mit der Säuberung endlich angefangen, nachdem er mit seinem Chef gesprochen hatte.

   The brewer has with the beer / the cleaning finally begun, after he had spoken with his boss.

   [low] Der Braumeister hat mit dem Buch / mit der Buchvorstellung endlich angefangen, nachdem er mit seinem Chef gesprochen hatte.

   The brewer has with the book / the book presentation finally begun, after he had spoken with his boss.

5. [high] Der Metzger hat die Würste / die Wurstherstellung vorsichtig begonnen, nachdem er den Hund gefüttert hatte.

   The butcher has the sausages / the sausage production carefully begun, after he had fed the dog.

   [low] Der Metzger hat den Schrank / den Umzug vorsichtig begonnen, nachdem er den Hund gefüttert hatte.

   The butcher has the cabinet / the move carefully begun, after he had fed the dog.

6. [high] Der Möbelpacker hat mit dem Schrank / mit dem Umzug vorsichtig weitergemacht, nachdem er eine Zigarette geraucht hatte.

   The mover has with the cabinet / the move carefully continued, after he had smoked a cigarette.

   [low] Der Möbelpacker hat mit den Würsten / mit der Wurstherstellung vorsichtig weitergemacht, nachdem er eine Zigarette geraucht hatte.

   The mover has with the sausages / the sausage production carefully continued, after he had smoked a cigarette.

7. [high] Der Regisseur hat mit dem Drehbuch / mit dem Casting pünktlich aufgehört, um seinen Zug nicht zu verpassen.

   The director has with the script / with the casting on time finished, not to miss his train.

   [low] Der Regisseur hat mit dem Aufsatz / mit dem Studium pünktlich aufgehört, um seinen Zug nicht zu verpassen.

   The director has with the essay / with the study on time finished, not to miss his train.

8. [high] Der Student hat mit dem Aufsatz / mit dem Studium pünktlich angefangen, um vor seinem Geburtstag fertig zu sein.

   The student has with the essay / with the study on time begun, to be done before his birthday.

   [low] Der Student hat mit dem Drehbuch / mit dem Casting pünktlich angefangen, um vor seinem

Geburtstag fertig zu sein.

The student has with the script / with the casting on time begun, to be done before his birthday.

9. [high] Das Geburtstagskind hat mit den Geschenken / mit der Feier sofort angefangen, obwohl seine Mutter nicht da war.

   The birthday child has with the presents / with the party straight away begun, although his mother was not there.

   [low] Das Geburtstagskind hat mit der Suppe / mit der Schicht sofort angefangen, obwohl seine Mutter nicht da war.

   The birthday child has with the soup / with the [work] shift straight away begun, although his mother was not there.

10. [high] Die Kellnerin hat mit der Suppe / mit der Schicht sofort angefangen, obwohl sie keine Lust hatte.

    The waitress has with the soup / the [work] shift straight away begun, even though she did not feel like it.

    [low] Die Kellnerin hat mit den Geschenken / mit der Feier sofort angefangen, obwohl sie keine Lust hatte.

    The waitress has with the presents / the party straight away begun, even though she did not feel like it.

11. [high] Der Informatiker hat den Code / die Fehlersuche umgehend begonnen, nachdem er eine große Tasse Kaffee getrunken hatte.

    The computer scientist has the code / the debugging immediately begun, after he had drunk a big cup of coffee.

    [low] Der Informatiker hat den Motor / die Reparatur umgehend begonnen, nachdem er eine große Tasse Kaffee getrunken hatte.

    The computer scientist has the engine / the repair immediately begun, after he had drunk a big cup of coffee.

12. [high] Der Mechaniker hat mit dem Motor / mit der Reparatur umgehend aufgehört, weil er mit anderen Dingen zu beschäftigt war.

    The mechanic has with the engine / the repair immediately finished, because he was too busy with other things.

    [low] Der Mechaniker hat mit dem Code / mit der Fehlersuche umgehend aufgehört, weil er mit anderen Dingen zu beschäftigt war.

    The mechanic has with the code / the debugging immediately finished, because he was too busy with other

things.

13. [high] Der Journalist hat mit dem Artikel / mit der Recherche ohne Überzeugung weitergemacht, weil es schon sehr spät war.

    The journalist has with the article / with the research without conviction continued, because it was already very late.

    [low] Der Journalist hat mit dem Kuchen / mit dem Verkauf ohne Überzeugung weitergemacht, weil es schon sehr spät war.

    The journalist has with the cake / with the sale without conviction continued, because it was already very late.

14. [high] Der Konditor hat den Kuchen / den Verkauf ohne Überzeugung vertagt, weil er zuerst die Weihnachtsplätzchen backen wollte.

    The baker has the cake / the sale without conviction postponed, because he wanted to bake the Christmas cookies first.

    [low] Der Konditor hat den Artikel / die Recherche ohne Überzeugung vertagt, weil er zuerst die Weihnachtsplätzchen backen wollte.

    The baker has the cake / the sale without conviction postponed, because he wanted to bake the Christmas cookies first.

15. [high] Der Professor hat mit dem Beispiel / mit der Vorlesung ohne zu zögern weitergemacht, weil er es eilig hatte.

    The professor has with the example / the lesson without hesitation continued, because he was in a hurry.

    [low] Der Professor hat mit dem Haus / mit dem Verkauf ohne zu zögern weitergemacht, weil er es eilig hatte.

    The professor has with the house / the sale without hesitation continued, because he was in a hurry.

16. [high] Die Maklerin hat das Haus / den Verkauf ohne zu zögern vertagt, weil es einfach nicht der richtige Moment war.

    The [female] broker has the house / the sale without hesitation postponed, because it simply was not the right moment.

    [low] Die Maklerin hat das Beispiel / die Vorlesung ohne zu zögern vertagt, weil es einfach nicht der richtige Moment war.

    The [female] broker has the example / the lesson without hesitation postponed, because it simply was not

the right moment.

17. [high] Der Gast hat den Kuchen / das Gespräch mit Freude begonnen, weil er sich schon lange darauf gefreut hatte.

    The guest has the cake / the meeting happily begun, because he had been looking forward to it for a while.

    [low] Der Gast hat die Mauer / den Aufbau mit Freude begonnen, weil er sich schon lange darauf gefreut hatte.

    The guest has the wall / the construction happily begun, because he had been looking forward to it for a while.

18. [high] Der Bauarbeiter hat mit der Mauer / mit dem Aufbau mit Freude weitergemacht, weil er einen guten Tag hatte.

    The construction worker has with the wall / the construction happily continued, because he was having a good day.

    [low] Der Bauarbeiter hat mit dem Kuchen / mit dem Gespräch mit Freude weitergemacht, weil er einen guten Tag hatte.

    The construction worker has with the cake / the meeting happily continued, because he was having a good day.

19. [high] Der Redakteur hat den Artikel / die Besprechung aus gutem Grund begonnen, obwohl er nicht wirklich daran interessiert war.

    The editor has the article / the meeting for a good reason begun, even though he was not really interested.

    [low] Der Redakteur hat das Pilzragout / den Frühjahrsputz aus gutem Grund begonnen, obwohl er nicht wirklich daran interessiert war.

    The editor has the mushroom ragout / the spring cleaning for a good reason begun, even though he was not really interested.

20. [high] Die Hausfrau hat das Pilzragout / den Frühjahrsputz aus gutem Grund vertagt, sie war nämlich krank.

    The housewife has the mushroom ragout / the spring cleaning for a good reason postponed, namely because she was sick.

    [low] Die Hausfrau hat den Artikel / die Besprechung aus gutem Grund vertagt, sie war nämlich krank.

    The housewife has the article / the meeting for a good reason postponed, namely because she was sick.

Appendix B

Below are the mixed-effect regression statistics when there are no covariates in the model. That is, these are the statistics of interest when word length, log-transformed word frequency, reading time at the previous region and presentation order were excluded as covariates.

Object Region
Type: $t = 1.149$, $p = 0.263$
Fit: $t = -1.651$, $p = 0.113$
Interaction: $t = -0.682$, $p = 0.499$

Object+1 Region
Type: $t = -0.680$, $p = 0.504$
Fit: $t = -2.632$, $p = 0.009$
Interaction: $t = -0.640$, $p = 0.522$

Verb Region
Type: $t = 0.244$, $p = 0.807$
Fit: $t = -0.108$, $p = 0.914$
Interaction: $t = -1.806$, $p = 0.074$

Verb+1 Region
Type: $t = -3.336$, $p = 0.001$
Fit: $t = -2.171$, $p = 0.040$
Interaction: $t = -0.671$, $p = 0.502$

Comparisons at the Verb Region:
high fit entity vs. low fit entity: $t = 0.182$, $p = .857$
high fit event vs. low fit event: $t = -2.407$, $p = .020$
high fit event vs. high fit entity: $t = -2.192$, $p = .037$
low fit event vs. low fit entity: $t = 0.257$, $p = .798$
